# Clinical development of immunotherapies for HER2^+^ breast cancer: a review of HER2-directed monoclonal antibodies and beyond

**DOI:** 10.1038/s41523-020-0153-3

**Published:** 2020-03-12

**Authors:** Ricardo L. B. Costa, Brian J. Czerniecki

**Affiliations:** Departments of Breast Oncology, H. Lee Moffitt Cancer Center and Research Institute, Tampa, FL USA

**Keywords:** Breast cancer, Metastasis

## Abstract

Human epidermal growth factor receptor 2–positive (HER2^+^) breast cancer accounts for ~25% of breast cancer cases. Monoclonal antibodies (mAbs) against HER2 have led to unparalleled clinical benefit for a subset of patients with HER2^+^ breast cancer. In this narrative review, we summarize advances in the understanding of immune system interactions, examine clinical developments, and suggest rationales for future investigation of immunotherapies for HER2^+^ breast cancer. Complex interactions have been found between different branches of the immune system, HER2^+^ breast cancer, and targeted treatments (approved and under investigation). A new wave of immunotherapies, such as novel HER2-directed mAbs, antibody drug conjugates, vaccines, and adoptive T-cell therapies, are being studied in a broad population of patients with HER2-expressing tumors. The development of immunotherapies for HER2^+^ breast cancer represents an evolving field that should take into account interactions between different components of the immune system.

## Introduction

The clinical classification of breast cancer is based on the presence of transmembrane receptors, namely estrogen and progesterone, along with the amplification or overexpression of the human epidermal growth factor receptor 2 (HER2) protein/oncogene^1,2^. HER2 is a tumor-associated antigen (TAA) that is overexpressed or amplified in ~25% of patients with breast cancer and correlates with poor clinical outcomes if not appropriately treated with HER2-targeted therapies^3^.

Passive immunotherapy with HER2-directed monoclonal antibodies (mAbs), such as trastuzumab and pertuzumab, in combination with chemotherapy has led to an improvement in clinical outcomes of patients with HER2-positive (HER2^+^) metastatic breast cancer (MBC), as these agents have been shown to improve median overall survival (OS) to as much as 57 months^4–6^. These improvements have been largely credited to the direct targeting of HER2 by mAbs, which leads to the downregulation of oncogenic intracellular pathways being triggered by HER2 activation through homo- and hetero-dimerization in the cancer cell membrane^7^.

Notwithstanding the success of HER2-targeted treatments, the challenge to treat patients with HER2^+^ MBC remains, as HER2^+^ MBC patients will experience disease progression while on treatment with approved HER2-targeted treatments. Standard of care for patients with HER2^+^ breast cancer is the antibody drug conjugate ado-trastuzumab (T-DM1), which has improved the median OS of patients with progressive disease by 4 and 7 months in the EMILIA and TH3RESA trial, respectively^8,9^. The immediate corollary to these data is the robust clinical predictive validity of the HER2 antigen, leading to renewed enthusiasm for developing new HER2-targeted treatments.

**Fig. 1:**
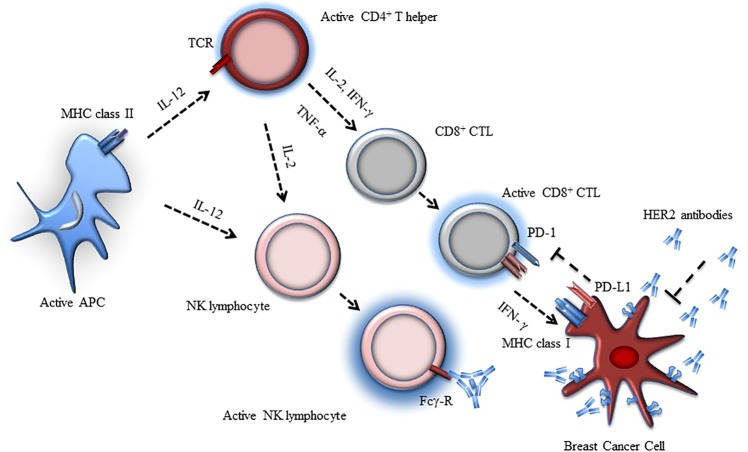
Depiction of immune response to breast cancer expressing HER2. HER2 antigen is processed by APC, leading to activation of cellular cytotoxic adaptive response (i.e., Th1 response mediated by CD4+ helper lymphocytes). CD8+ CTL can recognize the HER2 antigen through MHC I, leading to cytotoxic activity; breast cancer PD-L1 inhibitory activity and NK activation and function are also shown. The arrow depicts stimulatory effect.

In recent years, the use of immunotherapy to activate the adaptive branch of the immune system, through treatment with checkpoint inhibitors, such as PD-1 and PD-L1 inhibitors, has led to clinical benefit for the treatment of aggressive solid tumors^10,11^. As HER2 is a TAA, it can be targeted by a wide array of treatment strategies. To be effective, these treatments should ultimately lead to cytotoxicity by stimulating type 1 immunity (Th1). In this context, HER2-adaptive immune response is supported by both CD4^+^ and CD8^+^ T cells that secrete Th1 cytokines (Fig. 1)^11–15^.

Thus far, early phase clinical trials of new immune agents for the treatment of patients with HER2^+^ breast cancer have shown modest results. The phase 1b/2 KEYNOTE-014/PANACEA trial assessed the preliminary safety and efficacy of an anti-PD-1 mAb (pembrolizumab) combined with trastuzumab for the treatment of patients with HER2^+^ progressive MBC. Only 15% of the enrolled patients who showed PD-L1^+^ tumors (assessed in both cancer and immune cells) achieved a partial response, and there was no evidence of tumor response among patients in the PD-L1^–^ cohort^16^. These results suggest that the development of immunotherapies for HER2^+^ breast cancer will need to take into account more complex interactions between different components of the immune system and other treatment modalities.

Remarkably, there is mounting preliminary evidence showing that the antitumor activity of HER2-directed mAbs can also be attributed to the broad activation of the immune system, including both its adaptive and innate branches. For instance, FcγR-mediated activation of immune cells other than cytotoxic CD8^+^ T cells are necessary for antitumor activity^17^. In addition, the activation of CD4^+^ Th1 response against HER2 by HER2-primed dendritic cell (DC) vaccines have led to breast cancer tumor regression in an early phase clinical trial^18^.

Other treatments leading to immune-mediated cytotoxicity via other passive (e.g., adoptive T-cell transfer and cytokine administration) or active (e.g., HER2-directed vaccines) mechanisms are under development^6^. This narrative review aims to summarize the discoveries involving both passive and active immune therapies for the treatment of HER2^+^ breast cancer and highlight rationales for novel developmental therapeutic approaches.

## HER2-directed mAbs

Both trastuzumab and pertuzumab are HER2-directed mAbs. Trastuzumab is an IgG1 recombinant humanized mAb against the extracellular domain of HER2 (p185) and was developed to abrogate HER2 transmembrane signaling to cognate intracellular molecules. Pertuzumab binds to a different epitope of the HER2 dimerization domain than trastuzumab, preventing interactions with other receptors in the HER2 family that lead to cell growth inhibition^19–21^. The antitumor efficacy of HER2-directed mAbs has been largely attributed to their direct inhibitory action on the extracellular domain of HER2. Relevant interactions between HER2-directed mABs and the immune system, which lead to antibody-dependent cellular cytotoxicity, have been observed.

Albeit lacking clinical utility in routine clinical practice, the presence of polymorphisms in the immunoglobulin G fragment C receptors (FcγRs) present in cytotoxic cells (e.g., natural-killer [NK] lymphocytes, macrophages, and neutrophils) are associated with increased trastuzumab-mediated antitumor activity. FcγRIIa and FcγRIIIa are activating receptors present in these immune cells activated by the Fc portion of trastuzumab^22,23^. HER2^+^ mice models lacking common γ-chain receptors exhibit significantly less tumor mass reduction when treated with trastuzumab compared to mice models with preserved FcγRs^17,24^. In a study including 54 consecutive patients with HER2^+^ MBC who were treated with trastuzumab combined with taxane, it was found that the FcγRIIIa-158 V/V genotype (20%) was significantly correlated with an increased objective response rate (ORR) when compared with either 158 V/F (48%) or F/F (31%) genotypes (ORR, 82% vs. 42% vs. 35%, respectively; *P* = 0.03)^25^. Median progression-free survival (PFS) was not reached among patients with the 158 V/V genotype. Finally, there was a trend toward statistical significance in regards to ORR and PFS for the FcγRIIa-131 H/H genotype^25^. Significant correlations between improved complete pathological response rates and the FcγRIIIa-158 V/V genotype have also been reported among patients receiving neoadjuvant HER2-targeted treatment^26^.

In light of these findings, developmental therapeutics have gained momentum in fostering the clinical development of novel HER2-targeted mAbs. Margetuximab (MGAH22) is a chimeric anti-HER2 mAb with an Fc domain modified for improved binding to FcγRIIIa and lower affinity to its inhibitory FcγRIIB counterpart^27^. Antibody-dependent cytotoxicity assays showed enhanced activity of margetuximab against HER2^+^ cancer cells when compared with trastuzumab surrogates in donors with a low-affinity variant of FcγRIIIa (158F, homozygous or heterozygous)^27^.

The safety of margetuximab was assessed in a first-in-human phase 1 clinical trial that included 66 patients with metastatic progression of HER2^+^ solid tumors, 27 of whom had MBC^28^. Patients were treated with intravenous infusions of margetuximab at doses of 0.1–6.0 mg/kg for 3 out of every 4 weeks (Regimen A) or once every 3 weeks (10–18 mg/kg; Regimen B). All 66 patients were evaluated for safety. Infusion-related reaction and fatigue were the most commonly reported adverse events (AEs) attributed to margetuximab (18% and 14% of patients, respectively). Tumor reduction was observed in 11 out of 28 patients with breast cancer. All study participants had been previously treated with at least one HER2-targeted therapy. Given its favorable toxicity and tolerability profiles, margetuximab is under development in a dose-regimen of 15 mg/kg given every 3 weeks^29^. The clinical efficacy of margetuximab is being assessed in a phase 3 trial (SOPHIA trial, NCT02492711) comparing margetuximab combined with chemotherapy with trastuzumab plus chemotherapy. This trial includes patients with HER2^+^ MBC who progressed following HER2-targeted therapy. Preliminary results based on 536 patients with HER2^+^ MBC were presented^29^. Patients were randomized 1:1 to chemotherapy (i.e., choice of standard dose of capecitabine, eribulin, gemcitabine, or vinorelbine) with margetuximab or trastuzumab at standard doses. Margetuximab led to only a modest improvement in PFS over trastuzumab (median of 6 months vs 5 months; hazard ratio [HR], 0.76; 95% CI, 0.59–0.98; *P* = 0.033). Clinical outcomes were improved in FcγRIIIa 158F allele carriers (median PFS, 7 months vs. 5 months; HR, 0.68; 95% CI, 0.52–0.90; *P* = 0.005).

Another way to potentially enhance the antitumor efficacy of HER2-targeted mAbs is through the modification of the HER2 single-chain variable fragment (scFv) domain, which binds a target protein other than HER2^30^. Greene et al. reported the results of a breast cancer xenograft model, demonstrating the antitumor efficacy of a HER2 scFv and an IFN-γ engineered protein^31^. This new compound showed increased antitumor activity when compared to the 4D5 HER2-directed antibody. Clinical development of this agent is awaited.

## HER2-directed antibody drug conjugates (ADCs)

ADCs are being developed under the premise of producing increased cytotoxicity with a concomitant reduction in chemotherapy off-target AEs. ADCs are molecules composed of an antibody linked to a chemotherapy payload. These linkers can be classified into two main groups, cleavable and noncleavable, on the basis of the stability of their bonds with the two molecules. Noncleavable linkers require intracellular degradation before drug release and activity^32,33^. Aside from the obvious mechanisms of action inherent to the activity of the antibody and chemotherapy choices, ADCs are an attractive treatment modality for HER2^+^ breast cancer due to possible bystander effects^34^. As much as 15% of the HER2^+^ breast tumors present intratumoral HER2-expression heterogeneity and permeable cytotoxic payloads can exert cytotoxic effects to neighboring cells^35^. Nonetheless, the drug-antibody ratio should also be considered when developing ADCs. A higher payload per unit of antibody would lead to increased antitumor efficacy but also to greater toxicity. For example, a recently approved HER2 ADC (DS8201a) showed significant risk of serious AEs and treatment-related deaths in early phase clinical trials^36^.

T-DM1 is an ADC that is formed by the bonding of trastuzumab to a potent microtubule inhibitor emtansine (DM1)^37^. T-DM1 is an FDA-approved ADC for the treatment of HER2^+^ breast cancer. These two molecules are linked by noncleavable linkers (i.e., two disulfides) and have an average drug-antibody ratio of 3.5^33^. In a seminal phase 1 trial published in 2010, the safety and tolerability of T-DM1 was firmly established, as patients treated at the maximum tolerated dose (3.6 mg/kg every 3 weeks) level had a very low risk of grade 3/4 AEs^38^. Thrombocytopenia was the most common AE (∼13%), with no reported cases of serious bleeding reported. With overall response rates in early phase clinical trials ranging from 25 to 44% and a favorable toxicity profile and tolerability, T-DM1 continued to be developed in later phase trials^38,39^.

The EMILIA trial was a phase 3 study in which T-DM1 was shown to improve the median OS of patients with trastuzumab-resistant HER2^+^ MBC from 26 months to 30 months (*P* value not reported) when compared to capecitabine plus lapatinib, even in the presence of crossover treatment^8^. In another phase 3 trial (TH3RESA) in which T-DM1 was compared with the physician’s chemotherapy of choice, median OS was significantly longer among patients treated with T-DM1 (23 months vs 16 months, *P* = 0.0007, respectively)^9^. More recently, in the phase 3 KATHERINE trial, T-DM1 was shown to improve the 3-year disease-free survival rate from 77% to 88.3% (HR, 0.50; *P* < 0.001) when compared to trastuzumab for the treatment of patients with residual invasive HER2^+^ breast cancer after neoadjuvant therapy. T-DM1 was administered for a total of fourteen 3-week cycles after breast cancer surgical treatment^40^. T-DM1 is now FDA-approved for the treatment of HER2^+^ breast cancer in both the metastatic and adjuvant settings if residual disease is observed after neoadjuvant treatment.

DS-8201a is an ADC with a topoisomerase inhibitor payload (exatecan derivative) linked by a cleavable linker to a human IgG1 mAB. DS-8201a has an amino acid sequence homologous to trastuzumab and shows an ADC of 7.7^41,42^. DS-8201a has also shown activity against T-DM1–insensitive HER2^+^ breast cancer cell lines^42^. Preliminary results of a phase 1 dose-escalation trial of DS-8201a for the treatment of patients with metastatic breast and gastric cancers showed evidence of antitumor efficacy, as 43 out of 67 (64.2%) patients with HER2^+^ MBC who were previously treated with T-DM1 had a confirmed objective response to treatment^43^. Common AEs included nausea (73%), decreased appetite (59%), and vomiting (39%). It should be noted that two patients experienced interstitial lung disease (ILD) leading to death.

More recently, the anti-tumor efficacy of this DS-8201a was confirmed on a prospective, two-phase, dose-finding, phase 2 trial for patients with HER2^+^, heavily pre-treated, progressive MBC (DESTINY-01), leading to accelerated FDA approval of this novel agent on 23 December 2019^44^. The recommended dose of DS-8201 was 5.4 mg/kg given intravenously every 3 weeks until disease progression or intolerable toxicity. A total of 184 patients were treated with the currently approved dose regimen (5.4 mg/kg), and all patients had been previously treated with T-DM1 and pertuzumab, with a median number of five previous lines of treatment for breast cancer. The efficacy analysis of the study’s primary endpoint showed a clinically significant overall response rate of 60.9%. Notwithstanding the evidence of significant anti-tumor efficacy, treatment-associated grade 3/4 AEs and two deaths were observed. The two deaths were secondary to ILD. It is also noticeable that a total 25 patients (13.6%) had ILD, with a median time to ILD onset of 193 days (range of 42–535). Furthermore, grade 3 neutropenia was observed in 20.7% patients, which further suggests that patients should be monitored closely while on treatment. Currently, DS-8201a is being developed in two phase 3 confirmatory clinical trials for the treatment of patients with progressive unresectable or metastatic HER2^+^ breast cancer (DESTINY-Breast02 [NCT03523585] and DESTINY-Breast03 [NCT03529110], respectively). Furthermore, DS-8201a is under development in the DESTINY-Breast04 (NCT03734029), a phase 3 trial assessing this agent’s efficacy in treating patients with HER2-low breast cancer (i.e., IHC 1+ and IHC 2+/ISH- HER2-expression tumors).

To replicate the successful development of T-DM1 and DS8201a, other ADCs are currently under development for the treatment of HER2^+^ MBC. SYD985 is a cleavable ADC composed of a duocarmycin, a potent DNA-alkylating agent with two moieties (DNA-alkylating and DNA-binding) that bind into the minor groove of DNA^45^. The in vitro antitumor activity of SYD985 and T-DM1 was assessed using HER2-overexpressing cell lines (IHC3^+^), SK-BR-3 and UACC-893, with each cell line showing similar inhibitory potencies (IC50 6.9 and 15.7 ng/mL in SK-BR-3 and 54.1 and 35.9 ng/mL in UACC-893 for SYD985 and T-DM1, respectively)^45^. The in vivo activity of SYD985 was tested in cell line-derived xenograft models and in a breast cancer patient-derived xenograft model (PDX) with different HER2-expression statuses^46^. In BT-474 and MAXF1162 HER2 IHC3^+^ models, SYD985 showed significantly more antitumor activity than T-DM1. Remarkably, SYD985 was 3- to 50-fold more cytotoxic than T-DM1 in low HER2-expressing (2þ/1þ) cell lines. An open-label, randomized clinical trial comparing the efficacy of SYD985 with the physician’s treatment of choice for the salvage treatment of patients with HER2^+^ locally advanced or MBC is ongoing (NCT03262935).

XMT-1522 is another HER2 ADC with nanomolar potency, which is composed of a HER2 antibody (HT-19) and a dolaflexin platform; the latter is able to conjugate auristatin drug payload at a ratio of ~1–13^47^. In low HER2 mouse xenograft models, XMT-1522 was found to lead to complete tumor regression, which was not observed with T-DM1 treatment^48^. A phase 1 multiple-histology trial of XMT-1522 for the treatment of patients with HER2 1-3^+^ progressive MBC is ongoing (NCT02952729). Preliminary results showed no dose-limiting toxicities across any of the 6 planned dose levels and no treatment-related serious AEs. A total of 18 patients were evaluable for efficacy. One partial response was observed at the first restaging scans in a patient with HER2^+^ MBC who was previously treated with T-DM1. Two cases of stable disease were seen in HER2^+^ BC patients (with a duration of disease stability of 13+ and 12+ weeks)^49^.

The ADC ARX788 is a dolastatin analog (MMAF) that is coupled via a noncleavable linker to the HER2 IgG, with a mean drug-antibody ratio of 1.9, and has shown activity against HER2^+^ ovarian, gastric, and breast cancer cell lines^50^. Furthermore, ARX-788 was shown to induce regression in a trastuzumab-resistant–derived breast xenograft (JIMT-1) model and was found to be significantly more effective than T-DM1 at equivalent doses^50^. ARX788 is in its early stages of development in a multiple-histology phase 1 clinical trial. Only patients with HER2^+^ MBC are included, and all patients must have received prior treatment with trastuzumab (NCT03255070).

ADCT-502 is another ADC that is directed against human HER2 and is site-specifically conjugated to the highly cytotoxic pyrrolobenzodiazepine-based linker-drug tesirine^51,52^. The in vivo antitumor activity of ADCT-502 was compared to T-DM1 in both cell line-derived and PDXs^52^. For example, in a HER2 1^+^ fluorescent in situ hybridization-negative (FISH-) breast cancer PDX, ADCT-502 had increased dose-dependent antitumor activity when compared to T-DM1. Notwithstanding the favorable preclinical toxicity profile of this novel agent, a phase 1 clinical trial of ADCT-502 in patients with HER2-expression was terminated due to safety concerns (NCT03125200). The low drug-antibody ratio of 1.7 compared to other ADCs could lead to increased risk of off-target toxicity.

Another treatment strategy being developed is the use of biparatopic antibodies (BpAbs), which have the capability of binding two different nonoverlapping epitopes on the antigen, leading to increased ADC internalization and cytotoxicity in a broader range of HER2-expressing cancer cells. MEDI4276 is a novel compound composed of variable domain sequences of 39S (IG1 human HER2 mAb) and trastuzumab. MEDI4276 contains four antigen-binding units that target two different HER2 epitopes^53^. This chimeric immunoglobulin is able to block HER2-HER3 interactions, as it blocks both ligand-independent and ligand-dependent HER2 activation^54^. AZ13599185 is a tubulysin variant that inhibits microtubule polymerization during mitosis to induce cell death. MEDI4276 is an ADC produced from the conjugation of this aforementioned novel HER2 antibody with AZ13599185 via a noncleavable linker (i.e., maleimidocaproyl) to the HER2 antibody^54^. Preclinical results are so far promising, with MEDI4276 showing activity in trastuzumab-resistant HER2^+^ breast cancer cells^54^. The results of a phase 1/2 clinical trial of MEDI4276 for the treatment of patients with progressive HER2^+^ metastatic solid tumors are forthcoming (NCT02576548).

Moving forward, a possible differential toxicity profile of novel HER2 ADC should also be considered, as the toxicity of novel agents could be mediated by interactions with immune cells through the FcγR. For example, Uppal et al. suggested that T-DM1 inhibits megakaryocyte differentiation through DM1 intracellular accumulation in an FcγRIIa-dependent manner^55^. In addition, in an early phase clinical trial, thrombocytopenia was the dose-limiting toxicity of a bispecific mAB targeting HER2 and FcγRIII (2B1)^56^.

## Immune checkpoint inhibitors

In May 2019, based on results of a phase 3 trial (IMpassion 130), the FDA approved the use of atezolizumab in combination with paclitaxel protein-bound for the treatment of patients with PD-L1^+^ (ie, ≥1% expression by tumor immune cells) triple-negative breast cancer (TNBC)^57^. Among patients with PD-L1^+^ tumors, atezolizumab improved median PFS to 8 months compared with 5 months among patients treated with chemotherapy alone (*P* < 0.001). In the first interim analysis, the PD-L1^+^ exploratory analyses also showed an increase in median OS from 15.5 to 25 months, favoring treatment with atezolizumab. Updated OS results have been reported (data cutoff 1 January 2019), showing a median OS of 18 months compared with 25 months, favoring the study arm^58^. Collectively, these results suggest possible late antitumor efficacy. The clinical efficacy of immune checkpoint inhibitors for the treatment of patients with HER2^+^ breast cancer remains to be determined.

HER2-expressing breast cancer cells use the PD-1/PD-L1 checkpoint axis to evade cytotoxicity by immune cells^59,60^. It should be noted that PD-L1, a ligand for PD-1, seems to be constitutively expressed in a subset of HER2^+^ breast cancer patients^61^. Higher PD-L1 expression on HER2^+^ breast cancer has been shown to have a significant positive correlation with a higher tumor grade and tumor-infiltrating lymphocytes^62^. Preclinical studies in immune-competent mice showed that PD-1 and CTLA-4 inhibition improves the immune-mediated effects of HER2-targeted therapies through synergistic activation of CD8^+^ T cells^63,64^. These data provide a rationale for the clinical development of immune checkpoint inhibitors for the treatment of HER2^+^ breast cancer patients and the combination of these inhibitors with HER2-targeted therapies.

In the PANACEA trial, 15% of the patients who had PD-L1^+^ tumors had a partial response to treatment with pembrolizumab (anti–PD-1 mAb) combined with trastuzumab, and no responses were observed among patients with PD-L1^-^ tumors^16^. All patients had been previously treated with trastuzumab-based therapy. A similar correlation between checkpoint inhibitor antitumor efficacy and expression of PD-L1 was observed in a randomized phase 2 trial. In the KATE2 trial, patients with PD-L1^+^, HER2^+^ pretreated MBC had improved PFS when treated with T-DM1 combined with atezolizumab compared with T-DM1 alone (HR = 0.82 [95% CI: 0.55, 1.23]; *P* = 0.3332)^65^. In addition, in another phase 1 trial, 15 patients with HER2^+^ progressive MBC were treated with trastuzumab in combination with durvalumab (anti–PD-L1 mAb)^66^. None of the patients enrolled in this trial achieved a partial response, and four of the patients had stable disease. It should be noted that none of the patients had tumors harboring PD-L1 expression. Finally, results from the JAVELIN trial showed that no confirmed responses were observed among the 26 patients with HER2^+^ MBC treated with avelumab (anti–PD-L1 mAb)^67^. Other clinical trials exploring the efficacy of other immune checkpoint inhibitors are ongoing (Table 1). However, the results of trials published to date collectively indicate that mAbs targeting the PD-1/PD-L1 axis present low antitumor efficacy in unselected patients with HER2^+^ MBC and when administered to heavily pretreated patients. In parallel, it should be noted that PD-1/PD-L1 axis checkpoint inhibitors showed a low probability of tumor response (18%) among patients with heavily pretreated triple-negative MBC^68^. Despite having a modest initial efficacy signal, another checkpoint inhibitor (atezolizumab), when combined with chemotherapy in the first-line setting, showed a clinically meaningful benefit among patients with PD-L1^+^ triple-negative MBC. Clinical trials assessing the antitumor activity of atezolizumab in combination with HER2 mAbs and chemotherapy for patients receiving early line treatment for HER2^+^ breast cancer are ongoing (NCT03125928, NCT03726879).

**Table 1:** HER2-directed immunotherapy trials under development.

Compound	Classification	Study phase	Patient population	HER2 1-2+, non-amplified allowed, y/n	Lines of therapy, no.	Study population (*n*), no.	NCT ID
HER2-directed mab							
MCLA-128	HER2/HER2 mAb	2	HER2^+^ LAD or MBC	Yes	>1	120	03321981
GBR 1302	CD3 bispecific mAb	1/2	HER2^+^ LAD or MBC	No	>1	158	03983395
HER2-directed ADC							
RC48	HER2 ADC	1b/2	HER2^+^ LAD or MBC	No	>1	165	03052634
DS-8201a	HER2 ADC	3	HER2^+^ LAD or MBC	No	>1	600	03523585
DS-8201a	HER2 ADC	3	HER2^+^ LAD or MBC	No	>1	500	03529110
DS-8201a	HER2 ADC	3	HER2^+^ LAD or MBC	Yes	>1	540	03734029
RC48	HER2 ADC	1b/2	HER2^+^ LAD or MBC	No	>1	165	03052634
FS-1502	HER2 ADC	1	HER2^+^ LAD or MBC	Yes	>1	92	03944499
SYD985	HER2 ADC	2	HER2^+^ LAD or MBC	No	>2	345	03262935
ARX788	HER2 ADC	1	HER2^+^ LAD or MBC	Yes	>1	60	03255070
PD-1/PD-L1 immune checkpoint inhibitors							
Pembrolizumab	PD-1 mAb	1b	HER2^+^ LAD or MBC	No	>1	27	03032107
Pembrolizuab	PD-1 mAb	2	HER2^+^ stage I-III	No	0	174	03747120
Atezolizumab	PD-L1 mAb	1b	HER2^+^ LAD or MBC	Yes	>1	98	02605915
Atezolizumab	PD-L1 mAb	3	HER2^+^ LAD or MBC	No	>1	6000	03199885
Atezolizumab	PD-L1 mAb	2a	HER2^+^ LAD or MBC	No	0	50	03125928
Atezolizumab	PD-L1 mAb	3	HER2^+^ stage I-III	No	0	453	03726879
Atezolizumab	PD-L1 mAb	2	HER2^+^ MBC with CNS involvement	No	–	33	03417544
KN035	PD-L1 mAb	2	HER2^+^ LAD or MBC	NR	>1	59	04034823
Cytokine directed therapies							
IFN-γ	Th1 cytokine	2	HER2^+^ stage I-III	No	N/A	43	03112590
Tocilizumab	IL-6 receptor inhibitor mAb	1	HER2^+^ LAD or MBC	No	>1	20	03135171
Utomilumab	Receptor co-stimulatory of TNF mAb	1b	HER2^+^ LAD or MBC	No	>1	79	03364348
Utomilumab	Receptor co-stimulatory of TNF mAb	2	HER2^+^ LAD or MBC	No	>1	100	03414658
CAR-T-cell							
CAR T-cell	Anti-HER2 CAR T-cell NOS	1/2	HER2^+^ LAD or MBC	NR	>1	60	02713984

Trials listed at www.clinicaltrials.gov as of 22 August 2019.

*ADC* antibody-drug conjugate, *CAR-T* Chimeric antigen receptor T-cell, *CNS* central nervous system, *LAD* locally advanced disease, *mAb* monoclonal antibody, *MBC* metastatic breast cancer, *NCT* National Clinical Trial, *NOS* not otherwise specified, *NR* no response, *TNF* tissue necrosis factor.

Interestingly, Czerniecki et al. showed anti-HER2 CD4^+^ Th1 immunity to be a relevant component of HER2^+^ therapy, as loss of CD4^+^ Th1 responses correlated with poor prognosis and treatment responses^15^. The administration of a class II HER2 peptide-pulsed Type I polarized DC1 vaccine was shown to induce a strong anti-HER2 CD4^+^ Th1 response, with a pathologic complete response rate (pCR) among HER2^+^ ductal carcinoma in situ (DCIS) patients^69–71^.

Notably, patients with non-small cell lung cancer with increased circulating dysfunctional CD4 immunity (i.e., a baseline profile showing a low percentage of CD4-differentiated T cells) had no objective response to PD-1/PD-L1 blockade therapy in an observational study^72^. Receiver operating curve analyses showed a cut‐off value of >40% to identify objective responders with 100% specificity (*P* < 0.0003). Supporting this notion, preclinical data showed that the activation of anti-HER2 CD4^+^ Th1 immunity in TUBO mice models prior to immune checkpoint blockade leads to improved outcomes^73^.

## Cytokine-activated mediation

Passive activation of the cytotoxic branch of the immune system can be accomplished through the administration of Th1 cytokines. IFN-γ is known to stimulate both CD8^+^ and CD4^+^ Th1 response^31,74^. The combination of IFN-γ and an anti-HER2 antibody synergistically reduces tumor growth in HER2-expressing tumors^75^. Preliminary results of a phase 1 dose-escalation clinical trial showed that IFN-γ was tolerable for the treatment of patients with HER2^+^ MBC when combined with paclitaxel administered weekly and HER2-targeted agents, such as pertuzumab and trastuzumab^76^. Efficacy results from the dose-expansion cohort are forthcoming.

IL-12 is also known to increase IFN-γ levels in mice harboring HER2^+^ breast cancer cells^77^. IL-12 in combination with a HER2-mAb (4D5) showed antitumor activity through the activation of NK cells^78^. In a phase 1 trial, IL-12 was administered twice a week in combination with weekly intravenous infusions of trastuzumab to 15 patients with trastuzumab-naive MBC who either had IHC 2^+^ or 3^+^ HER2^79^. Fatigue and nausea were the most common all-grade AEs. One patient achieved a complete clinical response. The favorable toxicity profile of IL-12 when administered in combination with trastuzumab and paclitaxel was also observed in a small multiple-histology phase 1 trial^80^. These trials not only showed the favorable safety profile of IL-12 treatment combinations; they also showed that IL-12 can lead to increased IFN-γ production.

IL-2 has also been shown to increase IFN-γ production through the activation of NK cells in vivo^81^. As a proof of concept, in a phase 1 trial including 10 patients with HER2-expressing MBC (IHC 2^+^ and 3^+^), IL-2 showed an expansion of NK cells when combined with trastuzumab and was well tolerated; these results support further development of this treatment combination^82^. IL-2 in combination with trastuzumab was tested in a Simon 2-stage clinical trial for the treatment of patients with pretreated HER2^+^ MBC^83^. A ≥2-fold increase in the peripheral level of IFN-γ was detected in 8 out of the 13 patients treated, but no NK cell expansion was observed. No responses were observed, and 12 patients had disease progression with a median time to progression of 51 days (range, 29–326 days).

## HER2-directed vaccines

Active immunotherapy with HER2-directed vaccines is compelling, warranting further development for several reasons. Among these reasons is wider stimulation of the immune system, including its innate branch. Another reason is the potential for epitope spreading with the activation of immune response against other antigens and HER2 epitopes.

HER2 vaccination is currently under development using a number of different strategies, such as peptide-, protein-, DNA-, and whole cell- or cell lysate-based vaccines. Thus far, HER2 peptide (i.e., E75) and antigen-presenting cell vaccines (i.e., HER2-dendritic cells) are in more advanced stages of clinical development. A full review of the clinical and preclinical data supporting the development of the wide array of HER2 vaccine strategies is outside the scope of this review and has been reviewed elsewhere^6^.

## E75 vaccine

The E75 vaccine (i.e., E75 peptide [HER2 369-377]) is a 9-amino acid human leukocyte antigen– (HLA-) restricted peptide located in the HER2 extracellular domain. The E75 vaccine boosts immune response by activating CD8^+^ and CD4^+^ Th1 responses^84–88^. This vaccine has the limitation of HLA class I restriction, which decreases its ability to more broadly activate the immune system and limits the patient population in which it can be used.

Clinical studies on the safety and efficacy of E75 vaccines have been rationally developed in the adjuvant setting, which is a less immune-tolerate environment than the metastatic setting.

In these trials^89–91^, 108 women with high-risk HER2-expressing breast cancer were treated with E75^92^. After a prespecified follow-up time of 60 months, the disease-free survival rate for vaccinated women was 89.7% compared to 80.2% in the control group (*P* = 0.08). More recently, when compared with placebo, E75 vaccination failed to improve the disease-free survival of patients with HER2-expressing, high-risk breast cancer in a randomized phase 3 trial, leading to the conclusion that synergistic combinations may be needed^93^.

### HER2-dendritic cell vaccines

**Fig. 2:**
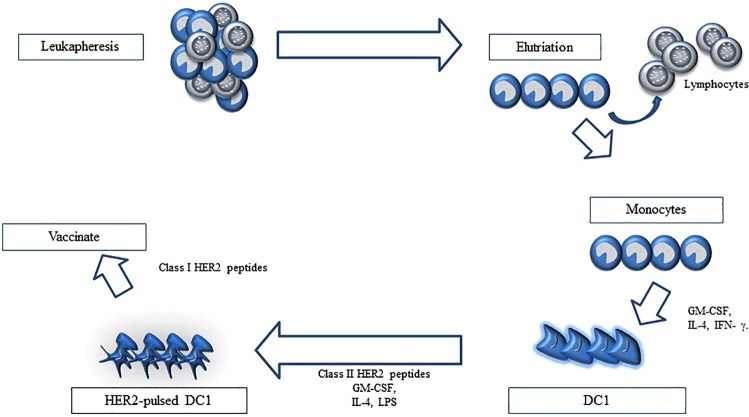
Preparation of HER2-pulsed dendritic cells vaccines. Preparation of the vaccine is a multi-step process starting with leukapheresis and countercurrent centrifugal elutriation to obtain the relevant monocyte pool. This monocyte pool is then treated with GM-CSF and IL-4, then pulsed with HER peptides and matured with IFN-γ and LPS for conversion into type 1 dendritic cells.

DCs are part of the antigen-presenting machinery, which costimulates both CD8^+^ and CD4^+^ T cells into Th1 responses against HER2^94^. In vivo models showed that HER2-pulsed DC vaccination boosts anti-HER2 Th1 immunity^95^. Autologous DC vaccines pulsed with both class I and II HER2 peptides (Fig. 2) have been more extensively studied in patients with HER2^+^ DCIS of the breast^96,97^. In an early phase clinical trial, a total of 54 patients with either HER2^+^ DCIS or invasive breast cancer received 6 weekly intratumoral and/or intranodal injections of DC1 vaccines pulsed ex vivo with 6 distinct MHC class II HER2^18^. Treatment was well tolerated and all patients completed the planned treatment. It is remarkable that as much as 81% of the patients showed peripheral blood activation of CD4^+^ and CD8^+^ Th1 response. Furthermore, pCRs were observed (DCIS, 28.6%; invasive breast cancer, 8.3%). This proof-of-concept trial showed that active stimulation of the adaptive immune system can lead to antitumor activity in HER2^+^ breast cancer. The efficacy of HER2-pulsed DC vaccines is being compared with WOKVAC vaccines (DNA Plasmid, which encodes for epitopes for HER2, IGFBP2, and IGF-1R) in a randomized phase 2 trial of patients with stages I-III HER2^+^ breast cancer (NCT03384914).

## Adoptive T-cell therapies

Several immunotherapy strategies are classified as being adoptive T-cell therapies. These strategies include the genetic modification of T-cell receptors via gene transfer technology that are able to recognize MHC I antigens with high affinity; the creation of chimeric antigen receptor T cells (CARs) by fusing a costimulatory specific antibody protein to the endogenous T-cell receptor; the infusion of ex vivo expanded tumor-infiltrating lymphocytes; and the infusion of peripheral ex vivo tumor antigen-primed and tumor antigen-expanded T cells. These modalities have been more extensively studied in patients with hematologic malignancies, and these studies have shown encouraging results for the treatment of subsets of patients with aggressive malignancies, such as acute lymphoblastic leukemia^98,99^. Currently, there are two FDA-approved CD19-directed CAR T-cell therapies: tisagenlecleucel and axicabtagene ciloleucel which are indicated for the treatment of (i) acute lymphoblastic leukemia and diffuse large B-cell lymphoma and (ii) relapsed or refractory B-cell non-Hodgkin lymphoma, respectively.

T cells against HER2 can be successfully expanded ex vivo in mice models and have shown evidence of antitumor activity^100^. One patient with HER2^+^ (Dako 3+) MBC was treated with autologous HER2_369-377_ T cells that were cocultured with HLA2-peptide-loaded DCs in a pilot trial^101^. There was evidence of tumor cell disappearance in the bone marrow but no penetration of T cells into the tumor.

HER2 CAR T cells containing CD28 costimulatory domain were administered to the central nervous system (CNS) of mice showing regression of HER2^+^ MBC in the CNS^102^. Clinical data of adoptive T-cell strategies are lacking for patients with HER2^+^ MBC, likely as a function of increased cost, needs for specialized centers for treatment development, and concerns related to off-target AEs secondary to the broad stimulation of the immune system against non-tumor specific antigens. Indeed, serous AEs have been reported with regard to HER2-directed CAR T-cell treatment as a function of acute cytokine release^101,103^.

## Immunotherapy-based combination strategies

As the complexities of interactions between different components of the immune system become known, other targets have started to emerge. For example, 4-1BB is a costimulatory receptor tissue necrosis factor (TNF) observed on CD8^+^, CD4^+^, and NK cells, which, when activated, leads to immune cell proliferation^104,105^. Utomilumab, a 4-1BB receptor IgG2 mAb agonist, is currently being developed in combination with trastuzumab or T-DM1 for the treatment of patients with advanced HER2^+^ breast cancer in a phase 1 dose-escalation trial (NCT03364348) and in combination with avelumab in a phase 2 trial (AVIATOR, NCT03414658) (Table 1). Preclinical data show that the combination of utomilumab and an mAb targeting the PD-1/PD-L1 axis leads to increased immune response^106^.

Modulation of the tumor microenvironment, including the tumor microvasculature, represents a further area of growing interest. It is currently not understood how tumor blood vessels can be altered to optimize drug delivery and facilitate immune cytotoxicity. Until now, antiangiogenic therapy has failed to show clinically significant improvements for the treatment of patients with HER2^+^ breast cancer using standard therapies^107^. Proangiogenic stimuli, such as increased vascular endothelial growth factor production, lead to immune evasion through a number of mechanisms, including the suppression of CD8^+^ T cells and DCs and the induction of PD-L1 expression by immune cells^108^. The latter mechanism supports the development of antiangiogenic agents in conjunction with immune checkpoint inhibitors.

Indoleamine 2, 3-dioxygenase 1 (IDO1) is an enzyme that catalyzes the metabolism of tryptophan in the tumor microenvironment, high levels of which mediate the inhibition of cytotoxic T cells via macrophages, DCs, and tumor cells^109–112^. In a TNBC model, IDO inhibitor D-1-methyl-tryptophan was shown to have in vivo antitumor activity. IDO overexpression was observed in a subset of HER2^+^ breast tumors (40%), which could be used to develop a synergistic treatment strategy, as observed in TNBC preclinical models^113–115^.

Toll-like receptors have also been shown to be associated with an adaptive immune response. Activation of Toll-like receptor 4, which is expressed by DCs, has been shown to increase antigen processing and cross-presentation in vivo^115^. Oligodeoxynucleotides containing CpG motifs activate Toll-like receptor 9, which has shown to active immune cytotoxicity in pre-clinical model^116^. In HER2^+^ breast cancer preclinical models, activation of Toll-like receptor 2 has been shown to augment trastuzumab-mediated cytotoxicity against HER2^+^ breast cancer cells^117^. Toll-like receptor agonists are being developed in combination with HER2-directed vaccines (NCT02276300).

## Immunotherapy for CNS disease

Through the course of their disease, patients with HER2^+^ MBC face a high risk of CNS involvement with an absolute risk (AR) as high as 40%^118–121^. Patients are usually treated with CNS-directed therapies, including surgery and or radiation therapy, and systemic therapies are offered upon CNS disease progression. Lapatinib (an oral tyrosine kinase inhibitor) in combination with capecitabine is commonly used for the treatment of patients with HER2^+^ CNS involvement, and phase 2 trial data support an objective CNS response of 66%^122^. Interestingly, there are emerging data showing that the antitumor efficacy of oral tyrosine kinase inhibitors may be secondary to not only direct abrogation of HER2 signaling but also to activation cytotoxicity Th1 immune response, as suggested by results observed in mice models^123^.

As oral HER2-targeted agents continue to show signs of clinical efficacy for the treatment of CNS disease, interactions between these agents and the immune system should be considered in the development of future treatments^124^.

Nonetheless, immunotherapies present a potential solution, as treatment with checkpoint inhibitors has shown CNS antitumor efficacy in patients with solid tumors^125^. Indeed, CNS breast tumor tissue analysis showed expression of PD-L1 in 53% of the cases (*n* = 84)^126^. A clinical trial assessing the antitumor efficacy of atezolizumab in combination with trastuzumab and pertuzumab for the treatment of patients with HER2^+^ breast cancer with progressive brain metastases is forthcoming (NCT03417544). The safety and preliminary efficacy of tremelimumab, a mAb against CTLA4, was assessed in combination with radiation therapy and trastuzumab in six women with HER2^+^ breast cancer with brain metastasss^127^. Two women had nonCNS disease control lasting over 12 weeks, and treatment was well tolerated. Further studies are needed to assess for a possible abscopal effect, which may be the cause of the observed improved outcomes.

## Discussion

HER2-directed mAbs have significantly improved the outcomes of patients with localized or metastatic HER2^+^ breast cancer. The clinical efficacy of second generation of treatments, including the ADC T-DM1, further supports HER2 as being a robust target for future treatment development. Immunotherapy has gained momentum for the treatment of a wide array of solid tumors using mAbs that target the PD-1/PD-L1 axis, immune checkpoint inhibitors. However, these agents have not shown clinically relevant results in unselected patients with HER2^+^ breast cancer. These findings, along with emerging clinical and preclinical data, suggest that more complex interactions between different components of the immune system, such as innate and adaptive branches, will have to be considered to develop treatments with clinically relevant efficacy.

From the preclinical standpoint, for optimal testing of new strategies, models will have to reconcile the ability to assess the preliminary efficacy of developing therapies with dealing with an immune-tolerant environment necessary for tumor growth. Nonetheless, the standardization of measures of immune activation and biomarkers predictive of benefit from therapy are lacking for both preclinical and clinical use. In addition, developing immunotherapies will need to account for synergistic interactions with chemotherapies. For a large subset of patients with HER2^+^ breast cancer, these interactions are an important component of treatment, as there are modest response rates associated with trastuzumab as monotherapy compared with combination treatments with chemotherapy (11% vs. 60%)^128,129^.

The clinical development of HER2 immunotherapies will have to surpass the challenge of the favorable efficacy to toxicity ratio of currently approved HER2-targeted agents used to treat MBC in the first-, second-, and third-line settings (overall response rates, 43–80%) and localized disease^130,131^. In the latter scenarios, another caveat is the possible additional or differential toxicity profile of new treatments aiming to broaden the activation of the immune system, such as the development of mAbs^132^. It should be noted that HER2-directed vaccines have shown favorable toxicity profiles, with negligible AR for grade 3/4 treatment-related AEs, but adoptive T cell–based therapies likely present with greater AR for AEs. This finding will need to be considered, along with increased patient treatment burden, for the development of future treatments^133^.

Notwithstanding these hurdles, activation of the immune system has the potential for self-sustained and prolonged antitumor activity. In exploratory analyses, it is remarkable that atezolizumab in combination with abraxane improved the survival rates of patients with PD-L1^+^ metastatic TNBC in the first-line setting when compared to chemotherapy alone (25 months vs 15 months; HR, 0.62)^57^.

**Fig. 3:**
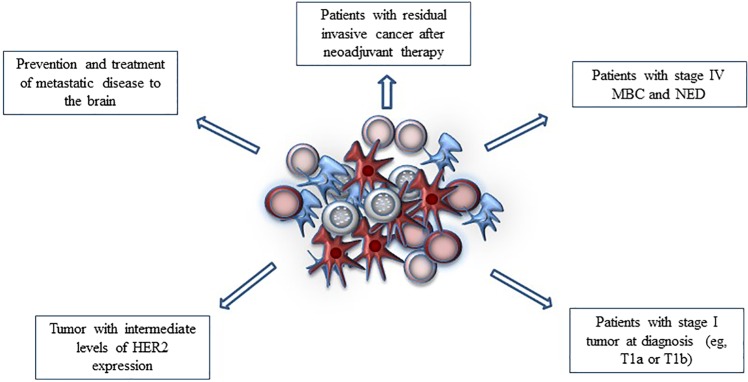
Possible areas of HER2 immunotherapy clinical development. Th1 response against HER2 breast cancer cells supported by CD8^+^, CD4^+^, NK lymphocytes, and DC.

It should be considered that HER2 immunotherapies could potentially be developed in a broader patient population, such as patients with an intermediate HER2-expression level who are not candidates for treatment with approved HER2 mAbs (e.g., HER2 2^+^, FISH^-^/dual in situ hybridization-negative). It is also more likely that HER2 immunotherapies are successful for the treatment of patients with low tumor burden as a function of having lower immune tolerance than patients with high tumor burden undergoing later lines of systemic therapies. There are numerous scenarios in which immunotherapies could be developed in the future with the goal of improving outcomes while deescalating chemotherapy to avoid significant AEs and improve clinical outcomes (Fig. 3). For instance, data suggest that CD8^+^ T-cell–mediated immunity can take place against tumors located in the CNS^134^, as evidenced in other solid tumors with brain metastases. The clinical relevance of developing strategies to prevent and treat HER2^+^ metastatic disease to the brain cannot be overstated, as these patients have a high risk of brain involvement (∼40%)^118–121^.

## Conclusions

HER2 overexpression/amplification is a biomarker predictive of strong correlations between improved clinical outcomes and treatments with HER2-targeted agents. Recent discoveries support the development of immunotherapies (both passive and active) aimed to activate different components of the immune system. In addition, passive therapies with novel mAbs and ADCs are in the advanced stages of development, as phase 3 clinical trial are ongoing.
